# Signals of stream fish homogenization revealed by AI-based clusters

**DOI:** 10.1038/s41598-018-34313-x

**Published:** 2018-10-29

**Authors:** Su-Ting Cheng, Wen-Ping Tsai, Tzu-Chun Yu, Edwin E. Herricks, Fi-John Chang

**Affiliations:** 1 School of Forestry and Resource Conservation, National Taiwan University, No. 1, Section 4, Roosevelt Rd., Taipei, 10617 Taiwan Republic of China; 2 Department of Bioenvironmental Systems Engineering, National Taiwan University, No. 1, Section 4, Roosevelt Rd., Taipei, 10617 Taiwan Republic of China; 3 Department of Civil and Environmental Engineering, The Pennsylvania State University, University Park, PA 16802-1408 USA; 4 Department of Civil and Environmental Engineering, University of Illinois at Urbana-Champaign, 205 N. Mathews Avenue, Urbana, IL 61801 USA

## Abstract

Risks of stream fish homogenization are attributable to multiple variables operating at various spatial and temporal scales. However, understanding the mechanisms of homogenization requires not only watershed-scale, but also exhaustive fish community structure shifts representing detailed local functional relationships essential to homogenization potentials. Here, we demonstrate the idea of applying AI-based clusters to reveal nonlinear responses of homogenization risks among heterogeneous hydro-chemo-bio variables in space and time. Results found that species introduction, dam isolation, and the potential of climate-mediated disruptions in hydrologic cycles producing degradation in water quality triggered shifts of community assembly and resulting structures producing detrimental conditions for endemic fishes. The AI-based clustering approach suggests that endemic species conservation should focus on alleviation of low flows, control of species introduction, limiting generalist expansion, and enhancing the hydrological connectivity fragmented by dams. Likewise, it can be applied in other geographical and environmental settings for finding homogenization mitigation strategies.

## Introduction

The homogenization process is often defined as the replacement of native species with a narrow geographic range by either native or non-indigenous species with a broad geographic range^1^. The process generally increases diversity at a watershed scale when the endemic species co-occur with the native generalists or non-native species^2^. Nonetheless, when extinction or extirpation of endemic species occurs^2,3^, the taxonomic diversity will be reduced at regional or global scales^4^.

One of the causes for homogenization has been documented as species expansion across their natural distribution boundaries^5^ stimulated and/or accelerated intentionally or accidentally by human activities^6^. Species expansion is mostly a result of species introduction, agriculture or aquaculture practices, and human transportation^7–9^. Other documented biotic homogenization is triggered by, and often co-involved with, large-scale environmental alteration such as dam construction, channel habitat modification, and climate change^10,11^. These modifications can, in some cases, produce expansion in the geographic range of some species, but may also lead to the extirpation of rare, localized, and endemic species^2,12^. Contribution to species change can be found in the construction of shipping canals that promote the movement of formerly confined fish and invertebrate species across historical geographical constraints^13^. Given that the local species composition is the result of interacting hydrologic, chemical, and biological (hydro-chemo-bio) factors, there should be a recognition that any existing ecological equilibria are due to simultaneous influence of a range of biotic and abiotic factors^14–16^. Consequently, identification of homogenization is challenging because multiple dynamic process are involved. There may be subtle interactions among species, the species present both respond to, and reflect, the influence of environmental variables, and human-induced change may influence ecosystems over local to global scales.

Extant ecosystems are complex with both structure and function influenced by species present, the interactions among species, and the response of each species to changes in local habitat and water quality conditions. Fish community assembly in watersheds is not random. Species presence and abundance are determined by the interacting factors in hydro-chemo-bio domains producing community structure that is known to change from upstream to downstream^17^. Although interacting factors provide a template for community assembly, there is much to be learned about homogenization processes.

With enhanced computational systems and big data mining techniques, there are new opportunities to illuminate factors influencing community structure. Using developments in artificial intelligence (AI), spatial and temporal drivers of homogenization have been revealed that are not seen using traditional statistical methods. For example, the self-organizing map (SOM) approach is an AI-based clustering^18^ that produces an unbiased and consistent analysis of community characteristics. Employing the SOM’s “shortest distance clustering principle” in a neighborhood function algorithm allows an unsupervised training and clustering procedure that preserves the properties of the input space and produces a self-forming topological map^19,20^. This map allows exploration of heterogeneous data relationships by data clustering and data mining, which then provides an unbiased analytical approach that can be used to evaluate environmental influences on aquatic communities^21^. Furthermore, this exploration of heterogeneous data opens a way to detect community change related to the complex environmental and biological interactions that operate at multiple watershed scales. The AI approach helps to delineate problems and inform mitigation directions^22–24^. In this paper, this approach provides a novel analytical procedure that can detect stream fish homogenization.

In this study, we aim to detect homogenization by finding indicators that identify possible causes of community change while recognizing emerging issues that may influence fish conservation and avoid homogenization. The main objective of this study is to develop a coherent framework for homogenization detection of highland stream fish communities in Taiwan considering site location in the watershed, habitat characteristics, water quality, and flow. The specific objectives are: (1) to explore the non-linear relationships among water chemistry, flow, and fish community structure in watersheds; (2) to examine the combined natural and anthropogenic influences on the local fish community structure; (3) to identify factors resulting in homogenization that can be related to a management goal of preventing the loss of endemic species.

## Materials and Methods

### Study area and data collection

**Figure 1:**
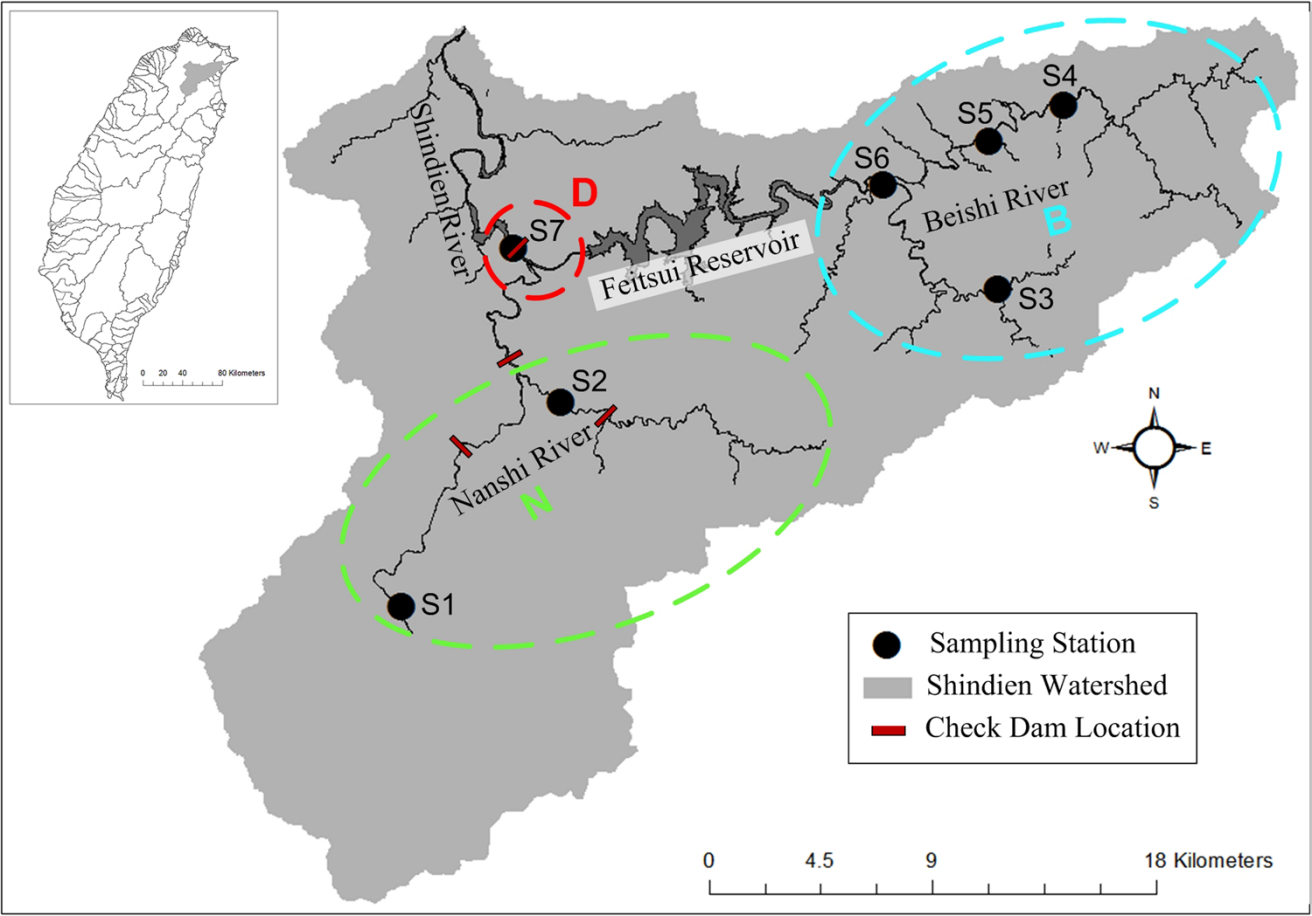
The Shindien River basin in northeast Taiwan features mountainous terrain in the headwaters of the Nanshi and Beishi rivers. Sampling locations (S1–S7) in the basin are shown. The Feitsui Reservoir and check dams alter flow and create barriers to fish passage.

Located in northern Taiwan, the Shindien (also known as Xindian) River watershed has a drainage area of 909.54 km^2^. The Shindien River has two major tributaries. The Nanshi River rises in the Chilan Mountain and flows mainly east approximately 82 km to the confluence with the Beishi. The Beishi River’s origin is also in mountainous terrain, flowing mainly west with a total length of 50 km to the confluence with the Nanshi. The Feitsui Reservoir is located on a downstream reach of the Beishi River. The confluence of the Nanshi and Beishi forms the Shindien River (Fig. 1).

The Shindien River watershed has been modified by both large reservoir (i.e., the Feitsui Reservoir) on the Beishi River, and small irrigation diversion dams on the Nanshi River. The check dams have local effects with a primary effect on downstream flow. The flow patterns in the watershed are typical of subtropical weather patterns and, recently, may reflect the influence of climate change. The Feitsui Reservoir was built in 1987. The check dams and agriculture-related diversion weirs have long been in place along the Nanshi River. In general, the Nanshi River, and the Beishi River upstream from the reservoir are typical highland streams with characteristics of headwaters that include small size, clear and cool water, and high gradient. The mainstem of these rivers grades to larger size, warmer water, and lower gradient. The upstream areas of the Shindien River, including the Nanshi and Beishi rivers, have been designated as “water-source protection areas” where landscape development is limited. The introduction of new species is not prohibited by law.

An examination of fish communities finds extensive endemism^25^ with the endemic species including the Formosan river loach (*Formosania lacustre*), Taiwan torrent carp (*Acrossocheilus paradoxus*), Formosan stripe dace (*Candidia barbata*), and Taiwan shoveljaw carp (*Onychostoma barbatulum*) among others. These endemics can be categorized as benthic-dependent, sediment-intolerant, and high-nutrient intolerant species^2^.

To investigate the possible mechanisms for fish community assembly, five sampling locations (S1, S3, S4, S5, and S6) were selected representing relatively natural river conditions with two locations (S2 and S7) subject to influences from check dams and the Feitsui Reservoir. Although agricultural activities and other human-related factors may influence all sampling locations, the designation as water resource protection areas has prevented new development of flow control structures. The sampling location selection was designed to minimize the effects from intense human activity. The presence of the Feitsui Reservoir is recognized for the change from flowing to standing water, a major influence on downstream locations due to flow alteration, and a fish passage barrier on the Beishi River. We aggregated long-term datasets (2005–2012) of fish sampling, daily flow, and water quality variables, which included water temperature (Temp, °C), pH, electrical conductivity (EC; μS/cm), suspended sediments (SS; mg/L), turbidity (Turb, NTU), biochemical oxygen demand (BOD; mg/L), dissolved oxygen concentration (DO; mg/L), ammonia-nitrogen (NH3-N; mg/L), and total phosphorus (TP; mg/L). Fish sampling had a reach-based design with locations related to fixed water quality sampling locations. Fisheries samples used for this analysis were from locations near water quality sampling sites and collected at the same time as water quality samples. Characteristics of daily flow data were extracted into monthly flow summaries similar to the Taiwan Eco-Hydrology Indicator System (TEIS)^26^. Fish surveys provided species distribution and abundance data.

### Model development

An initial assumption of our homogenization model considered habitat and water quality control of extant communities. We hypothesized that the difference of the fish species composition and abundance was attributable to site-specific habitat and water quality conditions. To identify unique conditions at different study sites, we compared the site-specific hydrologic and water quality data (e.g., flow regimes, Temp, pH, EC, SS, Turb, BOD, DO, NH3-N, and TP) by pairing the upstream stations in the Beishi River, Nanshi River, and the station downstream from the dam using a paired t-test employed by IBM SPSS Statistics 20, with a p < 0.05 providing a criterion for significant difference. The hydrology and water quality parameters identified as different were used. Where multiple sampling locations were available for the same tributary, we selected the upstream location to avoid bias from using similar stations in the analysis.

The pairwise similarity index (SI) was calculated using the Morisita-Horn method^27,28^ and used both richness and abundance of species to estimate the site similarity of two communities^29–31^. Following that, we gathered the t-test-selected water quality and flow regime parameters and calculated the paired-sites hydrology and water quality producing a total of 378 data metrics for use with SI values. Data was organized for similar time periods (i.e., same dates or a few days apart). All data in the metrics were individually normalized from 0 to 1. Joining biological with hydro-chemo data in the hierarchical ordination of SI made it possible to identify the abiotic and biotic relationships^32^.

We then employed the artificial intelligent technique, SOM, using MATLAB R2015b software, with an unsupervised learning and clustering method to nonlinearly interrelate hydrology, water quality, and biological data in an ecosystem analysis^33^. The SOM, with a topology-preserving neural network output, helps extract implicit patterns from high-dimensional multivariate input into a low-dimensional topological map^21,34^, where clustered patterns provide inter-relational features of input variables with a preserved data structure^35^. The SOM applied a competitive learning algorithm to organize training patterns into categories (clusters) to preserve the topological properties of the input variables. The constructed topological map was arranged on a hexagonal lattice using the Gaussian neighborhood function to display the clustered patterns so that the topology could be visualized, providing a system insight in data analysis.

**Figure 2:**
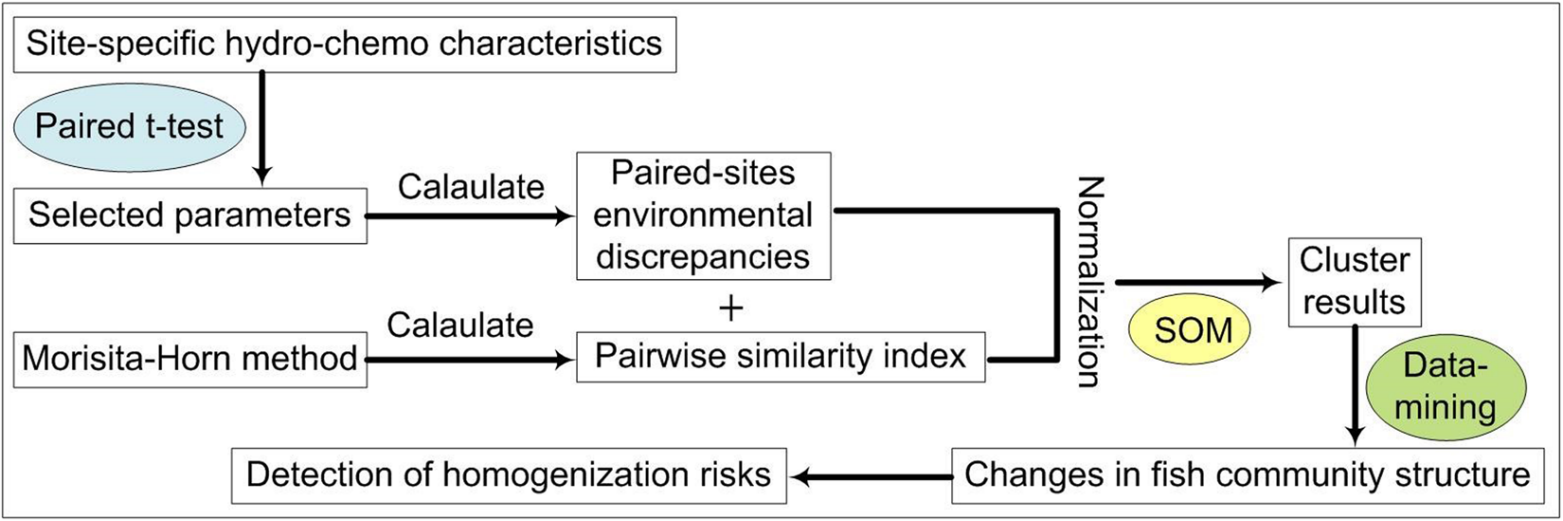
The overall analytical scheme uses a step-wise approach with a self-organizing map (SOM) supporting cluster analysis and data mining. The risk of homogenization considered data of Eco-Hydrology Indicator System (TEIS), water quality, and community similarity index for compared stations.

Lastly, a data-mining task was performed that inspected, in detail, the information provided by the SOM clusters. This data-mining also supported a return to the original data for a more comprehensive interpretation of the results. We grouped the paired-sites into BB (sites located in the Beishi River), NN (sites located in the Nanshi River), BN (one site located in the Beishi River and the other in the Nanshi River), BD (one site located in the Beishi River and the other downstream of the dam), and ND (one site located in the Nanshi River and the other downstream of the dam). We post-processed the calculation of species co-occurrence probabilities within each group in each cluster to investigate the potential for human-induced and climate-mediated changes on the risk of stream fish homogenization. The overall procedures for the methods used in this study are provided in Fig. 2.

## Results

### General cluster results

**Figure 3:**
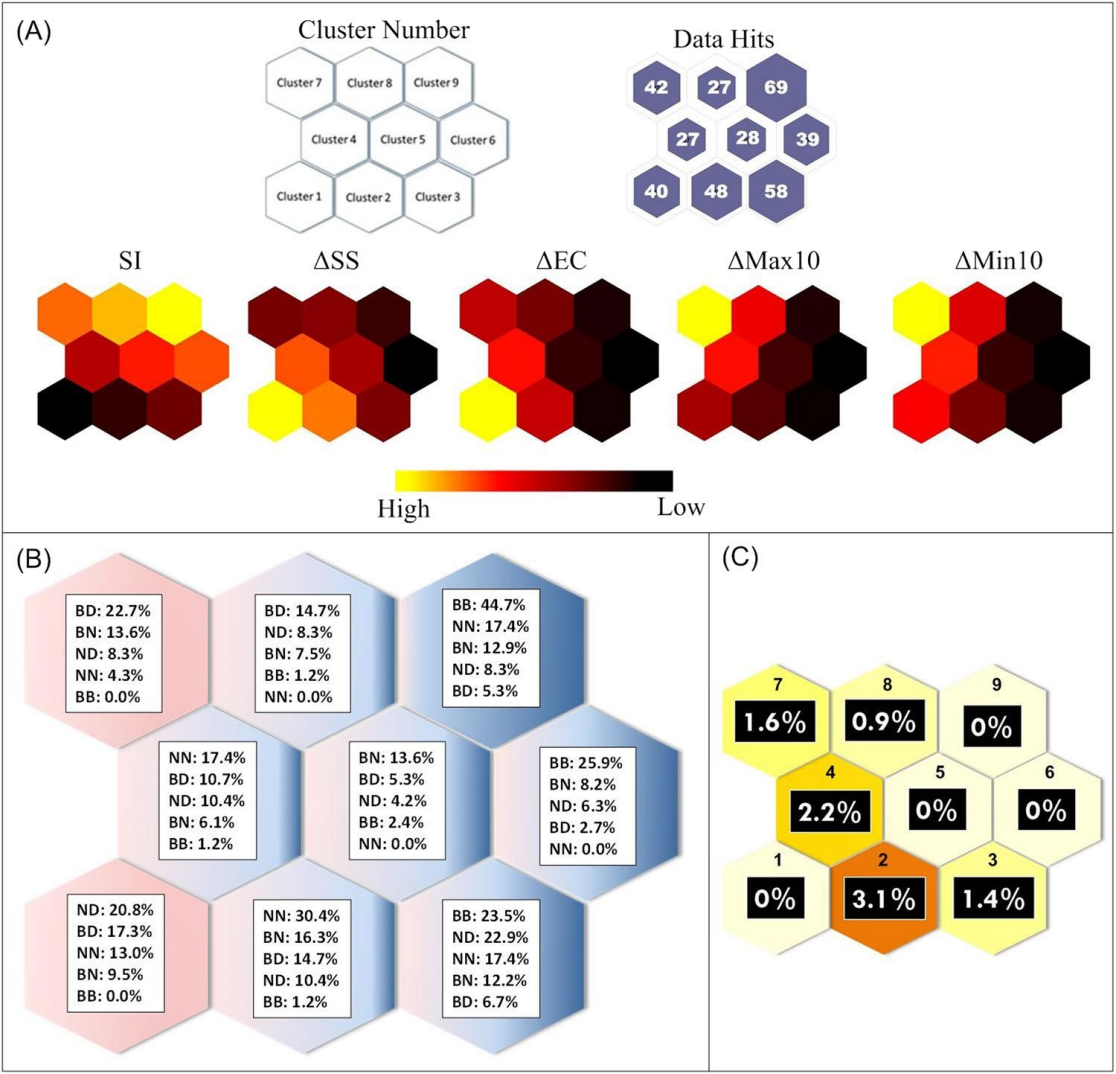
The results of SOM showing: (**A**) The topological maps of SOM associated with individual variables; (**B**) Spatial distribution of pairwise site locations; (**C**) Ratio of generalists (whether native or introduced) to endemics.

We first evaluated measured environmental parameters using the paired t-test. Among seven study sites, the paired t-test suggested that suspended sediment, electrical conductivity, maximum 10-day flow, and minimum 10-day flow were significantly different at these locations. Pairwise differences of suspended sediment (ΔSS), electrical conductivity (ΔEC), maximum 10-day flow (ΔMax10), and minimum 10-day flow (ΔMin10) were thus incorporated with the community similarity index (SI) to form parallel-input-metrics (a total of five variables) for the AI-based SOM. Displayed in SOM, the relationships among the parallel-input-metrics could then be arranged into five topological structures with nine clusters in each structure (Fig. 3A). The five topological structures represented the inter-relationship among the biological and environmental variables, while the nine clusters within each structure characterized the intra-relationships. Consequently, based on the “relationship distance” in the topological map, the SOM constituted ordination of distribution and gradient patterns representing non-linear relationships across the heterogeneous data inputs (Fig. 3A and Table 1)^35,36^.

**Table 1:** A summary of statistics for the five sets of AI-based clusters from each variable.

Cluster #	# of data	Statistics	SI	ΔSS	ΔEC	ΔMax_10_	ΔMin_10_
1	40	Ave.	0.12	80.01	36.56	56.14	35.09
		Range	0.00–0.49	0.00–299.80	0.00–65.00	3.99–211.59	4.02–58.74
		Std	0.13	105.74	16.53	48.14	16.85
2	48	Ave.	0.18	9.49	35.99	23.74	12.02
		Range	0.00–0.39	0.04–40.94	8.90–67.00	0.36–78.98	0.28–31.82
		Std	0.11	13.63	13.00	19.88	7.86
3	58	Ave.	0.20	3.96	12.02	18.07	9.78
		Range	0.00–0.37	0.02–41.35	0.00–31.00	1.99–67.01	0.10–27.82
		Std	0.11	8.88	9.12	14.05	6.92
4	27	Ave.	0.29	23.46	19.62	99.12	31.90
		Range	0.07–0.51	0.10–119.71	1.00–46.00	8.91–186.12	2.95–66.18
		Std	0.11	35.06	13.12	46.15	16.96
5	28	Ave.	0.46	2.80	31.58	24.74	9.42
		Range	0.37–0.58	0.00–14.80	9.00–81.00	0.42–61.95	0.10–23.76
		Std	0.06	3.88	15.39	18.20	5.88
6	39	Ave.	0.51	0.97	10.09	16.79	6.38
		Range	0.38–0.60	0.00–4.70	0.00–30.00	0.05–71.28	0.03–21.38
		Std	0.06	1.18	6.68	16.65	6.26
7	42	Ave.	0.56	17.98	18.74	148.75	60.61
		Range	0.11–0.97	0.02–117.07	0.00–78.00	59.33–289.20	29.86–101.81
		Std	0.22	35.51	18.58	61.09	18.79
8	27	Ave.	0.71	21.89	20.31	77.98	25.07
		Range	0.44–0.96	0.01–113.40	1.50–90.00	32.93–167.20	0.47–43.90
		Std	0.12	37.34	18.50	33.77	11.09
9	69	Ave.	0.78	4.32	15.67	17.07	8.35
		Range	0.62–0.99	0.00–108.06	0.00–59.00	0.22–42.32	0.12–24.73
		Std	0.11	16.84	12.55	11.47	6.55

A general decreasing trend of SI was found from clusters 9 to 1. Relating these results to the associated geographical information, we found a likely “distance effect” where SI values from distant sites had lower similarity when compared to closer sites (Tables 1 and 2). Linking with other parallel-inputs, we found that trend in SI was opposite from ΔSS and ΔEC (Fig. 3A). Correlation between trends of SI and those of the flow regime-related variables of ΔMax10 and ΔMin10 were not consistent in a horizontal comparison (i.e., clusters 1 to 3; 4 to 6; and 7 to 9) versus a diagonal comparison. Nonetheless, trends in ΔMax10 and ΔMin10 and their intra-related characteristics are similar (Fig. 3A) because they are driven by the continuous, unidirectional, upstream-to-downstream flow increase associated with river networks.

**Table 2:** Percentage of occurrence from each paired-sites location in each cluster.

Clusters Paired-Sites		1	2	3	4	5	6	7	8	9
NN	S1–S2	7.5%	14.6%	6.9%	14.8%	0.0%	0.0%	2.4%	0.0%	5.8%
BN	S1–S3	5.0%	8.3%	1.7%	7.4%	21.4%	2.6%	4.8%	7.4%	0.0%
	S1–S4	10.0%	12.5%	1.7%	7.4%	25.0%	0.0%	2.4%	7.4%	1.4%
	S1–S5	5.0%	8.3%	1.7%	0.0%	0.0%	0.0%	0.0%	0.0%	1.4%
	S1–S6	15.0%	16.7%	6.9%	7.4%	7.1%	0.0%	0.0%	0.0%	1.4%
	S2–S3	0.0%	0.0%	5.2%	3.7%	0.0%	10.3%	14.3%	7.4%	4.3%
	S2–S4	0.0%	0.0%	1.7%	3.7%	10.7%	7.7%	14.3%	14.8%	7.2%
	S2–S5	0.0%	0.0%	0.0%	0.0%	0.0%	5.1%	0.0%	0.0%	7.2%
	S2–S6	0.0%	4.2%	12.1%	3.7%	7.1%	5.1%	11.9%	3.7%	4.3%
BB	S3–S4	0.0%	0.0%	0.0%	0.0%	0.0%	2.6%	0.0%	3.7%	26.1%
	S3–S5	0.0%	0.0%	0.0%	0.0%	0.0%	2.6%	0.0%	0.0%	10.1%
	S3–S6	0.0%	0.0%	15.5%	3.7%	0.0%	17.9%	0.0%	0.0%	2.9%
	S4–S5	0.0%	0.0%	0.0%	0.0%	0.0%	5.1%	0.0%	0.0%	8.7%
	S4–S6	0.0%	2.1%	19.0%	0.0%	7.1%	17.9%	0.0%	0.0%	2.9%
	S5–S6	0.0%	0.0%	0.0%	0.0%	0.0%	10.3%	0.0%	0.0%	4.3%
ND	S1–S7	15.0%	6.3%	15.5%	11.1%	3.6%	2.6%	2.4%	0.0%	0.0%
	S2–S7	10.0%	4.2%	3.4%	7.4%	3.6%	5.1%	7.1%	14.8%	5.8%
BD	S3–S7	10.0%	2.1%	1.7%	14.8%	7.1%	2.6%	9.5%	11.1%	0.0%
	S4–S7	12.5%	8.3%	0.0%	7.4%	7.1%	0.0%	9.5%	22.2%	1.4%
	S5–S7	5.0%	0.0%	1.7%	0.0%	0.0%	0.0%	9.5%	0.0%	1.4%
	S6–S7	5.0%	12.5%	5.2%	7.4%	0.0%	2.6%	11.9%	7.4%	2.9%

Referring back to the pairwise data’s spatial information in the clusters, a spatial trend was identified in a location analysis. Clusters 9, 6, and 3 contained data primarily from the Beishi River (BB). Other pairs, mostly grouped in clusters 4 and 2, included sites in the headwater reaches of the Nanshi River (NN). Paired sites of BD (i.e., S7, downstream from the Feitsui Reservoir, with the headwater reaches of the Beishi Ricer) were mostly grouped in clusters 7 and 1; while ND (i.e., paired sites of S7 with those of the Nanshi River) were mostly grouped in clusters 3 and 1 (Fig. 3B).

Calculating the ratio of generalists, whether native or non-native, to endemics, produced a trend by clusters in a right-to-left direction (i.e., clusters 9-8-7; clusters 6-5-4; and clusters 3-2), and in an up-to-down direction (i.e., cluster 7-4-2), all representing ratios from low to high (Fig. 3C).

### Homogenization risk detection

**Figure 4:**
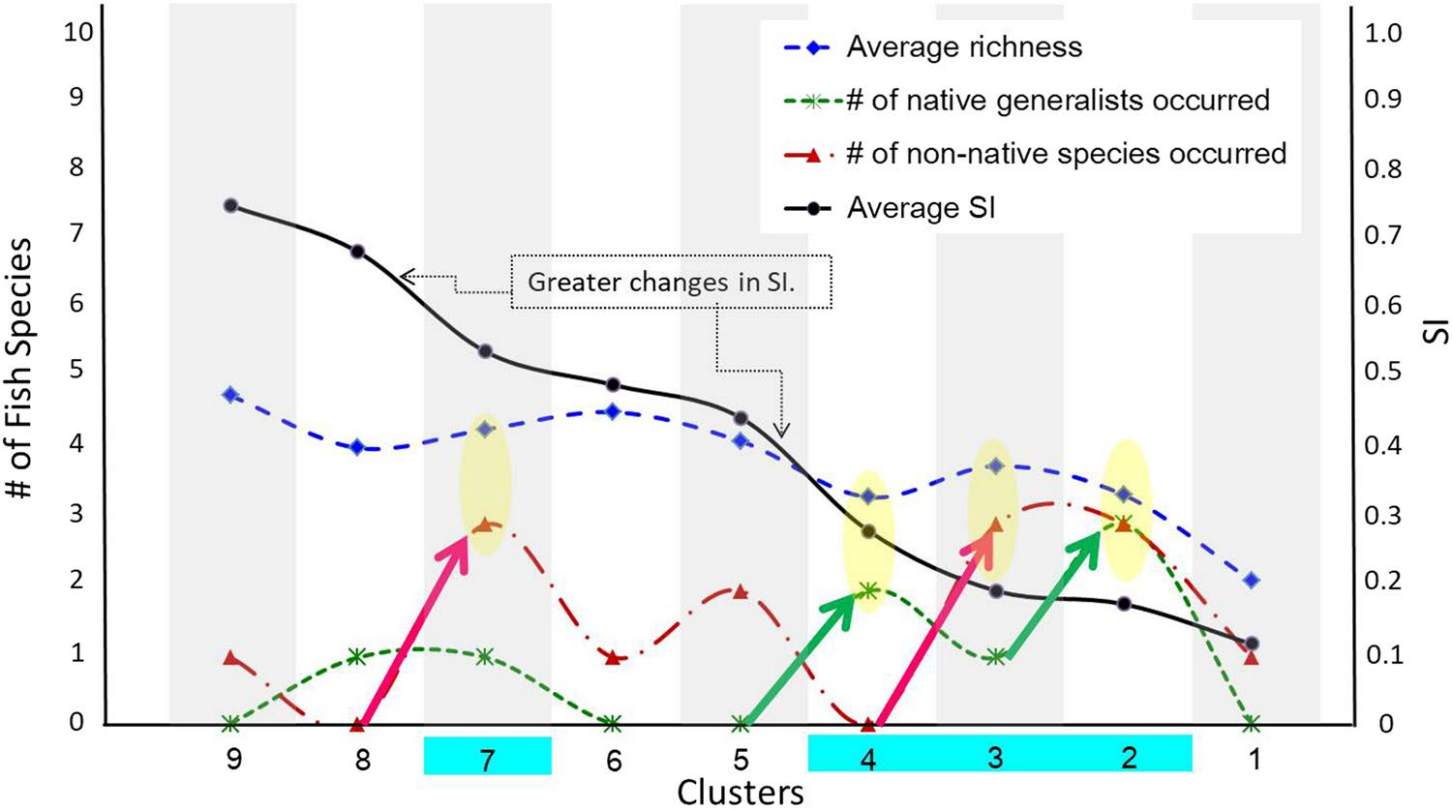
Decomposition of the relationships between SI, fish species richness, and clusters for the detection of homogenization signals.

As the value of SI decreased in clusters 9 to 1 (black line in Fig. 4), two greater changes of SI were identified in clusters 8 (SI = 0.71) to 7 (SI = 0.56) and clusters 5 (SI = 0.46) to 4 (SI = 0.29) (Fig. 4).

This decreasing trend corresponded with the increase in the total numbers of native generalists and/or non-native species present. The average number of non-native species (red dot-dash line in Fig. 4) increased in clusters from 8 to 7, while the average number of native generalists (green dash line in Fig. 4) increased in clusters from 5 to 4. Both the average number of native generalists and non-native species influenced the average richness (blue line in Fig. 4) where points circled in yellow corresponded to the increasing number of either native or non-native generalists. In contrast, clusters 9, 6, 5, and 1 had no generalists present. As a result, the AI-based clusters 7, 4, 3, and 2 (highlighted in light blue in Fig. 4) were considered to have homogenization signals reflecting relationships among SI, richness, and the assembly of local fish communities. These clusters were also associated with dam/reservoir locations.

**Figure 5:**
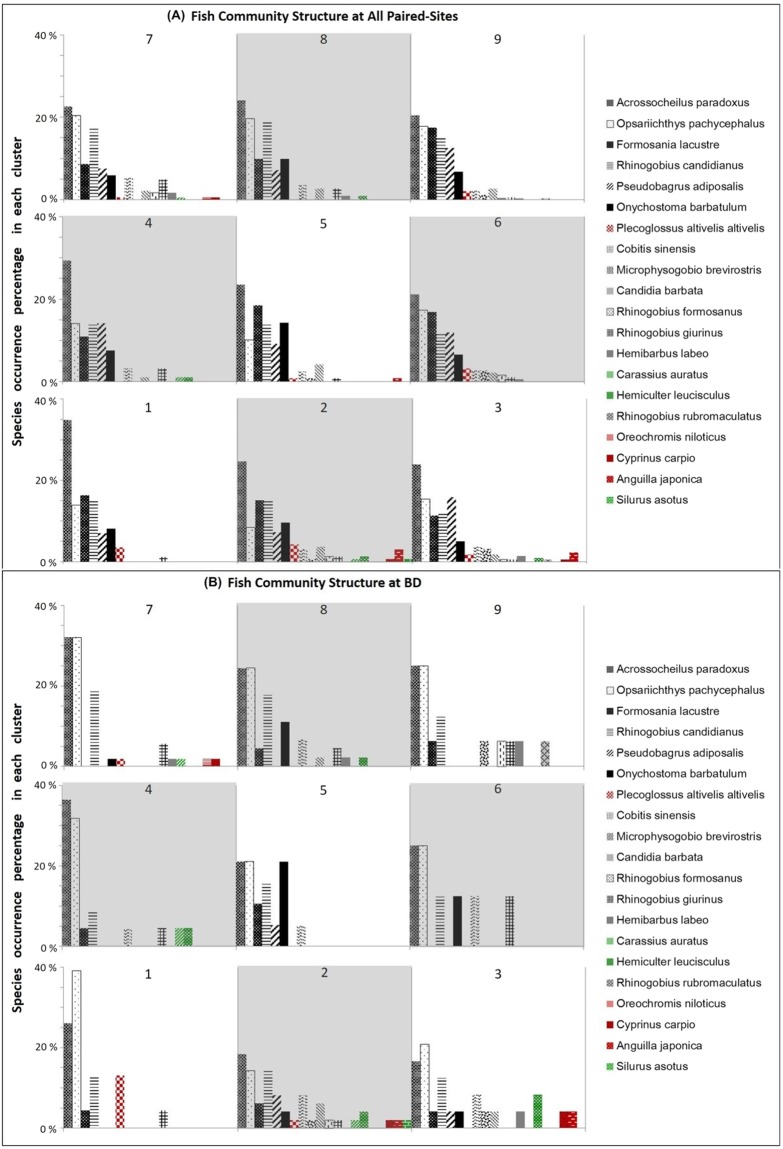
Using the single species occurrence information provided by AI-based clustering procedures, we identified the species composition in clusters and fish community structure using the percentage of single species occurrence in each cluster: (**A**) at all paired-sites, and (**B**) at BD. Green columns indicate native generalists and red columns indicate introduced species.

Further data-mining in the AI-based clusters focused on identification and interpretation of species composition identified the profile of fish community structure associated with environmental conditions (Fig. 5A). This analysis identified the native generalists or non-native species that could be related to homogenization risk in specific periods and locales. For example, native gobie species (*Rhinogobius formosanus* and *Rhinogobius giurinus*), skin-carps (*Hemibarbus labeo*), and spiny loaches (*Cobitis sinensis*) were present in cluster 7, where these species co-existed with the introduced species of sweet fish (*Plecoglossus altivelis altivelis*), tilapia (*Oreochromis niloticus*), wild common carp (*Cyprinus carpio*), and the goldfish (*Carassius auratus*; a native generalist, also known as native carp) (Fig. 5B). On the other hand, the river loaches (*Formosania lacustre*) and Formosan stripe daces (*Candidia barbata*) were not present in cluster 7 (Fig. 5A,B). In cluster 4, native species of gobies and spiny loaches, native generalists of minnows (*Hemiculter leucisculus*), and goldfish (*Carassius auratus*) co-occurred, but Taiwan shoveljaw carps (*Onchostoma barbatulum*), Formosan stripe daces (*Candidia barbata*), and Bagrid catfish (*Pseudobagrus adiposalis*) were not present (Fig. 5A,B). In cluster 3, three introduced non-native species of sweet fish (*Plecoglossus altivelis altivelis*), wild common carp (*Cyprinus carpio*), and Japanese eel (*Anguilla japonica*) were found to exist in most sites (Fig. 5A,B).

**Figure 6:**
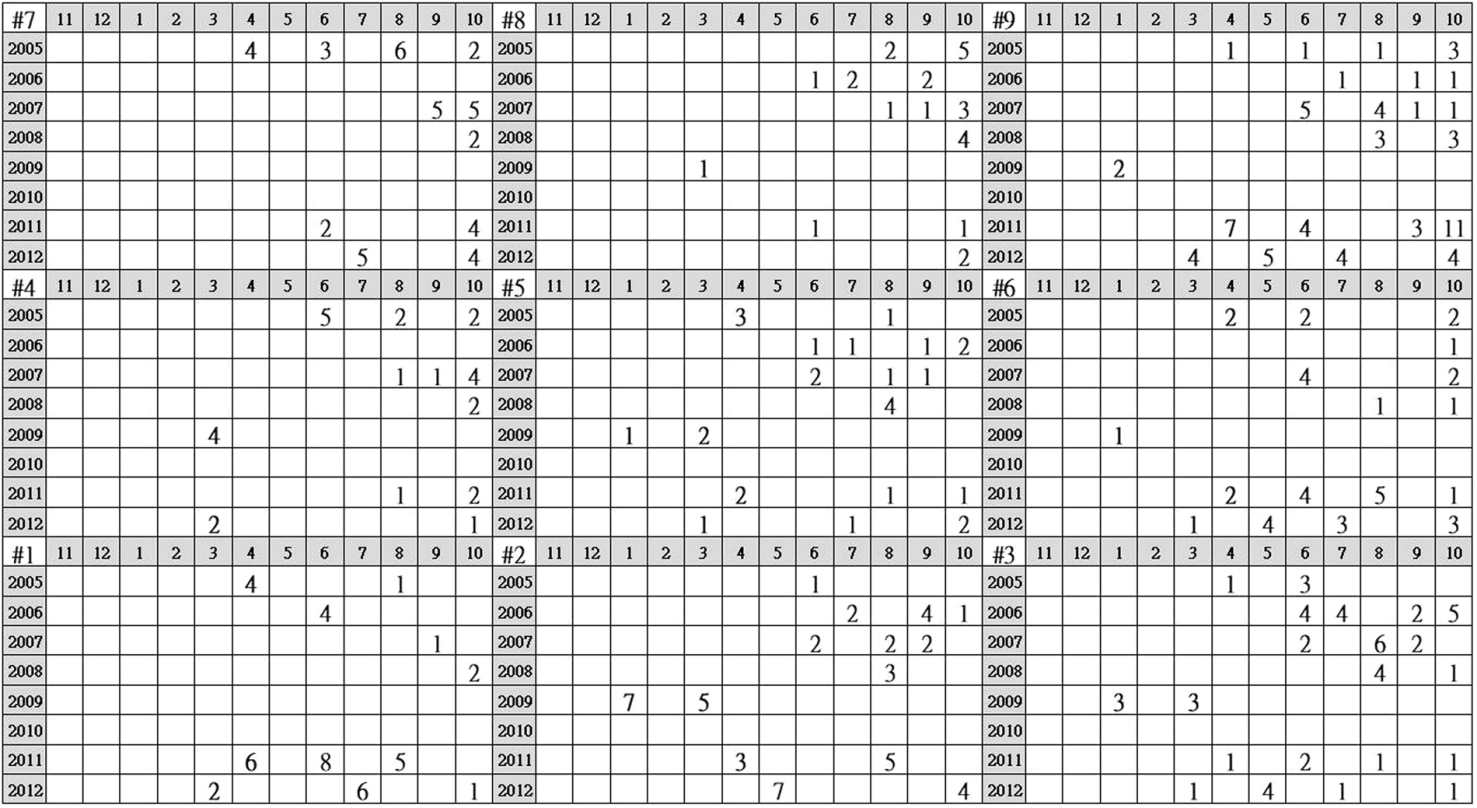
Temporal distribution among clusters. Numbers in each grid represent counts of the data metrics in the associated year and month.

Results found that cluster 2 possessed the strongest signal of homogenization risk across the nine clusters (Fig. 3C). Although cluster 2’s average SI was not the lowest, its ratio of generalists to endemics was the highest. The fish community in cluster 2 typically included three non-natives species, the sweet fish (*Plecoglossus altivelis altivelis*), wild common carp (*Cyprinus carpio*), and Japanese eel (*Anguilla japonica*), plus three native generalists including the minnow (*Hemiculter leucisculus*), native carp (*Carassius auratus*) and Chinese catfish (*Silurus asotus*) (Fig. 5A,B). In terms of the temporal distribution among clusters, we explored when samples in cluster 2 were collected and found that cluster 2 included mainly dry months or years, such as Jul. to Sep. 2006, Jun. to Sep. 2007, Aug. 2008, Jan. to Mar. 2009, Apr 2011, and May 2012 (Fig. 6).

## Discussion and Conclusion

This study explored the likelihood of homogenization revealed in indicators produced by an unbiased analytical approach that integrates physical, chemical, and biological data. Shifts in fish community structure and spatial and temporal changes in hydrology and water quality conditions were identified in the Shindien River watershed. Site analysis considered the expected environmental gradients occurring upstream to downstream in watersheds, the influence of large and small dams on river physical habitat, and the influence of watershed change on water quality parameters. Temporal considerations included flow variability in a subtropical setting and potential responses in rivers to watershed and climate change. An AI-based clustering method was used, which is different from the traditional clustering methods that are dependent on expert judgments for cluster identification. Using AI-based clustering with the SOM’s “shortest distance clustering principle” without supervision^34^, the AI-based clustering method is a powerful technique providing “unbiased” analysis to identify relationships among biological and environmental variables. Data-mining in clusters supported the identification of homogenization signals that were based on species composition, SI, water quality, flow, and site habitat descriptions^36^. In this study we demonstrated that analysis based on AI-based clustering considering site characteristics (e.g., water quality, flow, and fish community assemblages) in descriptive and integrated ways produced useful signals for homogenization detection. In addition, since the SOM allowed each topological structure to display patterns of individual variables, the clustered datasets reflected the ordination of site similarity associated with the input metrics^21^. The grouping of clusters allowed identification of similarities and differences between, and among, clusters providing insight into the effects of hydrology and water quality on site conditions^37^. In this study, AI-based clustering enabled the investigation of how water quality, flow, and geographical position related to fish community structure using homogenization signals. An improved understanding of factors influencing fish communities supports improvement in more comprehensive ecological conservation plans.

The analysis process initially suggested that possible controlling mechanisms, site hydro-chemo characteristics, and/or the cluster “distance effects” could be related to fish community assemblages. Clusters preserved the non-linear relationships among heterogeneous ecological, hydrologic and water quality metrics and showed linkages identified by the computational algorithm of SOM at multiple scales. Complex multivariate relationships in space and time were delineated by AI-based clusters incorporating the influence of environmental gradients and/or hydrological connectivity on fish community assemblages. Changes in SS and EC could be associated with SI. We found relatively low homogenization at paired-sites closer to each other or sites that had similar riverine environmental conditions. This result suggests that there is similar fish community structure in similar physical habitats with good water quality. At these locations, water quality conditions, in general, appeared to have stronger controlling effects than flow regime, but that was not always the case. Large differences of SS and EC between paired sites influenced fish community. However, at some sites where Taiwan endemic species were present, water quality and habitat were influenced by the general erosional characteristics of channels and geological settings rather than weather- or flow-induced change related to degradation (Fig. 3A).

The analysis found that flow regime influenced fish community^38^ with mechanisms operating on macro-habitat scales^39^. The ΔMax10 and ΔMin10 identified the annual pattern of typhoons and droughts in subtropical climates that were related to the SI. In particular, droughts were related to a greater likelihood of homogenization. The clustering results supported a finding that a lower SI and a higher ratio of generalists-to-endemics often occurred during dry seasons. This condition was not identified during longer term drought conditions where natural flows were maintained in protected watersheds. The maintenance of natural flows benefited endemic species that were adapted to natural flow regimes. The increased diversity of endemic species produced higher SI values leading to the site similarity identified by clusters. Basically, the pairwise data associated upstream areas which grouped sites with similar hydrologic and water quality conditions in both dry and rainy seasons. In many cases, the unidirectional natural flow regimes balanced the heterogeneity of the inhabitant communities that determined the status of the aquatic ecosystems^40^ through dynamically inter-related river flows and the variability of water quality across the river networks.

Decomposing and transforming the information revealed in the original datasets among clusters opens up several opportunities to better understand historical environmental-ecological issues in defining watershed conditions. For instance, the AI-based clusters provide a partial historical picture of site similarity prior to the construction of the reservoir. The pairwise comparison of data from the two rivers (i.e., Beishi and Nanshi Rivers) found that these rivers share 12 endemic species that were identified in the 9 site-related clusters. As seen in the co-occurrence of the same fish species found in both the rivers, if there were no dams, the dispersal distances could be as long as the distance across the two tributaries to most of the upstream reaches. The research results could also act as solid evidences to support the historical habitat connectivity before this connectivity was destroyed by the reservoir constructions.

Besides the hydrologic and water quality contribution to habitat conditions, this study found that dams strongly influence homogenization^10,11^. Site comparisons found indicators of homogenization in sites downstream and upstream from dams independent of SI values. Dam influence on riverine habitats is well known^25,41^. Our analysis suggests that homogenization is a likely effect of small dams and larger reservoirs. The hierarchical cluster of sites similarity assisted in the assessment of bio-geographical boundaries and identified endemic species susceptible to homogenization. Fish species showing the influence of dams are the Taiwan shovel-jaw carp (*Onychostoma barbatulum*) and certain upstream families, such as *Balitoridae*, *Cobitidae*, *Cyprinidae*, *Bagridae*, and *Gobiidae*. These species are particularly vulnerable to dams because dam structures obstruct movement. Changing riverine, running water habitats to reservoir, standing water habitats have caused effects beyond the restriction of free movement of fish upstream and downstream. Reach isolation also increases competition for limited resources. We know that identifying complex species interactions is not possible using the data used for community structure determination so we recognize that other study designs will be needed to support identification of these mechanisms of community assembly and maintenance.

An important outcome from this analysis is identifying how information can be extracted from data sets not developed to support the assessment of specific issues, such as homogenization. Assuming that samples reflect a local fish community, which has reached dynamic equilibrium reflecting the influence of exogenous environmental variables, the co-occurrence probability of endemics is a homogenization signal. We found that in comparison of data in clusters, higher SI values usually had higher numbers of endemic species, which suggests a lower homogenization potential for extirpation due to species intolerance. This was confirmed in clusters with lower SI values where endemic species numbers were lower due to watershed disturbances^11^. These disturbances that increased homogenization potential were identified as the introduction of non-native fish species as well as the presence of native generalists. Analysis showed connections between lower SI and the increased ratio of introduced species and/or native generalists to the endemic species. Endemics were less competitive in reaches with introduced or native generalists when SS or EC variations were higher. This is likely the results of generalists’ wider tolerance, allowing generalist species to establish themselves and grow populations while endemics had low populations or were extirpated. This was evident in degraded habitats, in streams closer to the dams, and/or during droughts.

Currently, none of the endemic fish species in the Shindien River were listed as threatened species. We worry that massive modifications, either from the warming climate or from human activities, could dramatically speed up the homogenization^42^, producing the extirpation/extinction of the endemic species. While the environmental determinants continue favoring generalists, whether introduced or native, the fish community assemblage will lose endemics and fish community diversity. With no understanding of the speed of homogenization, we are concerned that favoring generalists or introducing non-native species will be particularly destructive for endemics, which will face both environmental and ecological challenges. Consequently, it is important to identify homogenization potentials so that endemics can be carefully managed to maintain sustainable populations where extant conditions presently meet the needs of endemic species. Identifying the importance of endemic species in ecosystems and knowing that endemics are a key indicator of homogenization suggests a number of management approaches. Promoting establishment of any known invasive species should be avoided. Care must also be taken to encourage generalist species. Although non-native and generalist species are components of present fish communities, these species can out-compete endemics, leading to extirpation or extinction of endemics. Intrinsically we argue that although the value of richness has been used as a useful indicator for the ecological status^25,43,44^, increasing richness does not prevent homogenization because introduced species reduce ecological “space” for endemics and produce a high homogenization potential. As a result, homogenization potential should be the focus of fisheries and environmental management rather than an emphasis on more traditional measures of community health and stability.

In conclusion, with new analytical tools available, management should focus on homogenization of fish species communities when dealing with the non-linear and reach to regional issues in fish community sustainability. It is possible to identify homogenization signals in existing datasets. Through an AI-based cluster analysis that supports partitioning and comparing, as well as post-processing for nested information at integrated spatial-temporal scales, the AI-based clustering method provides critical insights to detect the homogenization signals for fishery conservation at further resolution from reach- to watershed-scales. Accordingly, we suggest that to better conserve endemics maintenance and management should focus on identifying multi-variable relationships and mechanisms among natural and anthropogenic environmental changes, and carefully consider species diversity and/or community structure. Under such complex circumstances, the AI-based clustering is a useful technique to present an unbiased analysis helping recognize homogenization causes and direct effective mitigation solutions.

## Data Availability

River flow data can be accessed at the Taiwan Water InfoShare & Exchange, Water Resource Agency of Ministry of Economic Affairs, R.O.C. at http://wise.wra.gov.tw/. Water quality data can be accessed at Environmental Water Quality Information, Environmental Protection Administration of Executive Yuan, R.O.C. (Taiwan) at https://wq.epa.gov.tw/Code/Station.aspx?Area=1140&Water=River&Languages=en. Fish distribution data that support the findings of this study are available from the Taipei Water Management Office, Water Resource Agency of Ministry of Economic Affairs, R.O.C., but restrictions apply to the availability of these data and are not publicly available. Data are however available from the corresponding author (F.-J. Chang) upon reasonable request and with permission of the Taipei Water Management Office.
